# Osteosarcoma Pathogenesis Leads the Way to New Target Treatments

**DOI:** 10.3390/ijms22020813

**Published:** 2021-01-15

**Authors:** Isabel Fernandes, Cecília Melo-Alvim, Raquel Lopes-Brás, Miguel Esperança-Martins, Luís Costa

**Affiliations:** 1 Medical Oncology Department, Hospital Santa Maria, Centro Hospitalar Universitário Lisboa Norte, 1600 Lisbon, Portugal; cecilia.alvim.moreira@gmail.com (C.M.-A.); raquellopesbras@gmail.com (R.L.-B.); miguelmemartins@campus.ul.pt (M.E.-M.); luiscosta.oncology@gmail.com (L.C.); 2 Luís Costa Lab, Instituto de Medicina Molecular, Faculdade de Medicina da Universidade de Lisboa, 1600 Lisbon, Portugal; 3 Sérgio Dias Lab, Instituto de Medicina Molecular, Faculdade de Medicina da Universidade de Lisboa, 1600 Lisbon, Portugal

**Keywords:** molecular targets, osteosarcoma, pathogenesis

## Abstract

Osteosarcoma (OS) is a rare condition with very poor prognosis in a metastatic setting. Basic research has enabled a better understanding of OS pathogenesis and the discovery of new potential therapeutic targets. Phase I and II clinical trials are already ongoing, with some promising results for these patients. This article reviews OS pathogenesis and new potential therapeutic targets.

## 1. Introduction

Osteosarcoma (OS) is thought to originate from mesenchymal stem cells and is the primary malignant bone tumor that most commonly affects children, adolescents, and young adults. OS preferentially develops in rapidly growing bone, especially in the metaphysis of long bones, like the distal femur, proximal tibia, or humerus [1,2]. Disease etiology remains unclear and controversial, but multiple associations have been made between OS development and race, gender, age, genomic alterations, and certain exposures such as to ultraviolet and ionizing radiation or chemical agents like methylcholanthrene, asbestos, or chromium salts [1,3,4]. The current management strategy for newly diagnosed OS includes neoadjuvant chemotherapy (ChT) followed by surgical removal of the primary tumor and all clinically evident metastatic disease with adequate margins, plus the addition of adjuvant ChT after surgery [2]. ChT protocols have traditionally included doxorubicin, cisplatin, ifosfamide, and methotrexate, although data raised in nonrandomized studies have questioned the use of methotrexate in OS treatment [2,5,6]. After surgery, it is important to assesses necrosis in the resected tumor. Patients with at least 90% of necrosis in the primary tumor after ChT have a better prognosis than patients with less necrosis [7]. These data have been critical in the attempts to identify patients who may benefit from therapy modifications, but new data revealed that, despite increasing the number of good responders, neoadjuvant ChT intensification did not alter overall survival, diminishing the prognostication value based on histologic response [8]. Another topic of discussion was the survival effect of changing post-operative ChT based on histologic response. The EURAMOS-1 trial addressed this question by randomizing good and poor responders to standard ChT or intensification therapy with pegylated interferon (IFN) alfa-2b. No significant differences in overall survival were identified between treatment arms in this study, showing that the degree of tumoral necrosis should not be used to guide decisions about postoperative systemic treatment [9].

Prior to 1970, localized OS treatment primarily relied on surgical resection, with 5-year survival rates below 20% [10]. However, with the developments in neo-adjuvant ChT, these have increased to 66–82% over the past 40 years [10]. Despite all these developments, OS remains a poor-prognosis disease, with 5-year survival rates of only 20% in patients with metastases, and is also a high-burden disease, which significantly impacts the patients’ quality of life and the community, as it affects patients in the prime of their lives, often with disabling surgery and long rehabilitation periods [11]. Therefore, the development of new treatment approaches is essential, and the understanding of OS molecular biology and potential therapeutic targets is crucial for that development. The aim of this work is to review molecular targets in OS considering new molecular biology developments and address emerging therapeutic modalities for this type of tumor.

## 2. Osteosarcoma (OS) Pathogenesis

The difficulties in OS biology research are related to the complexity of the OS genome, low incidence of this tumor, and significant biologic differences between OS subtypes. Different OS neoplastic clones develop, during tumor growth, from normal cells that earn the first cancer-promoting mutations to start tumor formation [12]. Various cell types along the osteogenic lineage have been suggested as cell-of-origin. Not only the cell-of-origin, but also their derived cancer stem cell (CSC) subpopulations are strongly affected by both environmental and epigenetic elements and it is then simple to understand that molding and shaping the OS-CSC environment and niche is the strategy behind different recently postulated therapies [12].

The intricacy and complexity of karyotypes and the nature of changes in multiple genes and cell pathways characterize, specifically, OS among sarcomas. The resulting significant genetic instability of operating system cells leads to the development of several different cell types within the same tumor, with consequent changes in cellular behavior. These changes may be responsible for the aggressiveness of cancer cells and result in the emergence of resistance to ChT treatment [11]. Understanding the main mechanisms of OS molecular pathogenesis, discussed below in this article, can help to unravel novel therapeutic approaches.

Several chromosomal and genetic syndromes, like Li-Fraumeni or hereditary retinoblastoma, have been linked to OS as well as 6p21, 8q24, and 12q14 chromosome amplifications and loss of heterozygosity of 10q21.1, described as the most common genomic alteration in OS [13]. Mutations in both the p53 or Rb suppressor genes have also been implicated in OS pathogenesis, but without evidence that they impact tumor behavior [14].

Transcription factors such as the activator protein 1 complex, found to be significantly upregulated in high-grade OS and associated with propensity to metastatic development, may play a future role as potential therapeutic targets [15]. Amplification of Myc, a transcription factor that exerts its effects in the nucleus promoting cell growth and division, has been involved in OS pathogenesis and resistance to chemotherapeutics [16]. OS cells have the capacity to develop and secrete a range of growth factors that exert autocrine and paracrine effects. Abnormal production and expression of these factors can lead to accelerated cell proliferation. Transforming growth factor (TGF)-β influences a wide variety of cell processes such as differentiation, proliferation, apoptosis, and matrix production, and is found be significantly overexpressed in high-grade compared with low-grade OS [17]. IGF (insulin-like growth factor)-I and IGF-II are growth factors frequently overexpressed in OS. They bind to specific receptors such as the IGF-1 receptor (IGF-1R), activating the PI3K and MAPK transduction pathways [18]. Parathyroid hormone-related peptide (PTHrP) and its receptor have also been implicated in OS progression and metastasis development, with PTHrP conferring OS chemoresistance by blocking signaling via p53 [19].

Another relevant factor in OS molecular pathogenesis is the resistance of OS cells to anoikis. Anoikis consists of a type of apoptosis that specifically takes place when cells lose their attachment to a basement membrane or matrix. It is particularly important in OS given the propensity of this tumor’s clones to detach from the matrix components and metastasize. The pathways involved in this process are intricate and comprise interactions between integrin signaling, Rho GTPases, PI3 kinase, and PKB/Akt activation, along with many key components of the intrinsic and extrinsic apoptosis pathway [20,21].

Tumor angiogenesis is essential for sustained OS growth and metastatic development. Vascular endothelial growth factor (VEGF) is a very well characterized pro-angiogenic factor, promoting endothelial cell proliferation, migration, and blood vessel maturation. These actions are potentiated via phospholipase Cγ, protein kinase C, and the c-Raf-MEK-MAPK cascades [22]. Readjustment of the actin cytoskeleton, crucial for endothelial cell migration, develops via phosphorylation of T cell-specific adapter and interaction with Src, a protein kinase [23]. VEGF also upregulates matrix metalloproteinase, responsible for breaking down extracellular matrix, inducing antiapoptotic factors, and releasing other pro-angiogenic factors such as platelet-derived growth factor (PDGF) or angiopoietin 1 [24,25].

As stated before, matrix metalloproteinases play an important role in extracellular matrix degradation, opening the possibility of the invasion of surrounding tissues. Another important mediator of this process is the urokinase plasminogen activator (uPA) system, which once activated cleaves plasminogen to plasmin. An inverse relationship between uPA levels and survival has been shown, and in vivo models have shown that downregulation of this system results in reduced primary tumor growth and fewer metastases [26].

Ultimately, bone invasion relies on interactions between osteoblasts and osteoclasts. Osteoclasts play a main role as bone-resorbing cells, and significant osteolysis exhibited in some OS cases is the direct consequence of the increased osteoclastic activity. Throughout the first stages of OS invasion, growth factors like TGF-β are liberated from the degraded bone matrix and have a direct action on OS cells, stimulating the release of PTHrP, interleukin(IL)-6, and IL-11 [27]. These cytokines stimulate osteoclasts, facilitating further invasion and release of pro-resorptive cytokines. Osteoclast pathways of differentiation, maturation, and activation constitute possible therapeutic targets, since the inhibition of bone resorption at the tumor–bone interface may conduct to reduced local OS invasion. The crucial role played by the receptor activator of nuclear factor kappa-Β ligand (RANKL) in osteoclast function makes it a particularly compelling target. Osteoprotegerin, a soluble decoy receptor for RANKL, vigorously suppresses osteoclast differentiation, both in vitro and in vivo [28].

Several signaling pathways have been associated with tumorigenesis in OS such as the Wnt and Notch pathways. Lately, deregulation of microRNAs (miRNAs)—non-coding RNAs that participate in post-transcriptional regulation of gene and protein expression—have shown a role in carcinogenesis, as discussed further below.

OS immunogenicity is closely linked with the intrinsic immunogenic properties of cancer clones, while the activity patterns of different immune cells that are part of the OS microenvironment influence the nature of the elicited immune response [29].

OS is typically associated with high levels of chromosome structural variations. Among these are rearrangements resulting from chromothripsis (20–89%) and mutation clusters known as kataegis (50–85% of cases), which result in a significant degree of genomic instability, with a predicted elevated burden of antigens and neoantigens that may provide immunogenic potential in OS [29,30]. Interestingly, the high levels of genomic rearrangements and moderate point mutation burden are associated with low levels of predicted neoantigen expression and are not associated with increased immune infiltrate levels [29]. Rather than evoking a vigorous immune response, this genomic complexity seems to contribute to multiple immune-suppressive mechanisms that may represent targets for novel therapeutic approaches [29].

Moreover, research has shown that poor tumor infiltration by immune cells, low activity from available T-cells, lack of immune-stimulating neoantigens, and multiple immune-suppressing pathways all combine to dampen response to immunotherapy in this tumor.

## 3. Potential Molecular Targets

OS is characterized by genomic complexity and significant heterogeneity. Its molecular biology and growing knowledge of tumor pathogenesis has allowed for the identification of several potential molecular targets including IGF-1 receptor antagonists, human epidermal growth factor receptor 2 (HER2)/neu receptor blockers, RANKL and bisphosphonate inhibitors, tyrosine kinase receptors targets, tyrosine kinase Src antagonists, VEGF inhibitors, immunomodulating agents, mammalian target of rapamycin (mTOR) inhibitors, MiRNA therapeutic targets, and signaling pathways and epigenetic regulators such as Hedgehog (Hh), Wnt/β-catenin, and Notch [30]. The latest research efforts are to identify OS patients more likely to benefit from immunotherapy.

### 3.1. Insulin-Like Growth Factor (IGF)-1 Receptor Antagonists

The IGF pathway is important to prevent apoptosis during normal development and stress or disease situations in several types of cells including osteoblasts and OS cells [30]. Consistent IGF-1R, IGF-1, and IGF-2 expression has been described in OS cell lines and clinical samples, suggesting that stimulation of the IGF autocrine system may be important for OS proliferation [31]. Small tyrosine kinase inhibitory molecules investigated in OS phase I clinical trials include linsitinib (OSI-906) in combination with erlotinib and BMS-754807 [32]. Additionally, monoclonal antibodies are also being studied in this condition (Table 1):

- Figitumumab (CP-751.871) is a monoclonal antibody in investigation for OS treatment, with a phase I clinical trial currently recruiting patients with advanced solid tumors (including bone sarcomas) to assess safety and tolerability of the antibody in combination with other drugs [30]. The R1507 phase II study, which includes an OS group, is currently underway.
- Phase I of a multicenter study with cixutumumab (BMI A12) in the treatment of young patients with relapsing or refractory OS and other solid tumors has been completed, with definition of the maximum tolerated cixutumumab dose [30].
- Robatumumab (SCH717454) showed extensive in vivo activity against solid tumors in the Pediatric Preclinical Testing Program, with complete response in two OS xenografts. Phase II studies of this antibody in the treatment of patients with recurrent Ewing’s sarcoma and OS concluded that, although IGF-1R remains an attractive treatment target, additional research is needed to identify responders and/or means to achieve durable remissions in order to successfully exploit IGF-1R signal blockade as a therapeutic target [33].

### 3.2. HER2/neu Receptor Blockers

Several research groups have investigated HER2 expression in OS, with divergent results [34]. A Cooperative Children’s Oncology Group (COG) study, which ended in October 2007, evaluated the addition of trastuzumab to standard ChT in patients with metastatic OS with tumors overexpressing HER2 (Table 1). Results suggested that trastuzumab could be safely delivered in combination with anthracycline-based ChT and dexrazoxane, but its therapeutic benefit remains uncertain. A more definitive assessment of trastuzumab’s potential role in OS treatment would require a randomized study of patients with HER2-positive disease [34].

### 3.3. PDFG (Platelet-Derived Growth Factor) Inhibitors

It has been shown that PDGF acts as a mitogenic factor for mesenchymal cells and OS cell lines [35]. It has also been demonstrated that PDGF-AA and platelet-derived growth factor receptor (PDGFR)-α co-expression in OS correlates with poor prognosis, highlighting PDGFR as a potential therapeutic target.

Imatinib, a c-Kit and PDGFR inhibitor, inhibited in vitro PDGF-mediated growth and apoptosis in OS cell lines [35]. In spite of this uplifting preclinical evidence, phase II data from the COG study of imatinib use in children with refractory or relapsing solid tumors including OS failed to show activity against OS as a single agent at conventional doses (Table 1) [35].

### 3.4. Receptor Activator of Nuclear Factor Kappa-Β Ligand (RANKL) and Bisphosphonate Inhibitors

These agents alter the tumor microenvironment and their potential benefit in OS is still being investigated outside of clinical trials [30].

A 2010 study by the Memorial Sloan Kettering Cancer Center assessed survival, event-free survival, and durability of orthopedic reconstruction of the addition of pamidronate, a second-generation bisphosphonate, to ChT (cisplatin, doxorubicin, and methotrexate) in 12-monthly doses. This phase II study concluded that pamidronate could be safely incorporated into ChT without effectiveness impairment for OS treatment, and could also improve limb reconstruction durability (Table 1). However, it is still to be determined whether this addition translates into a survival benefit [36].

### 3.5. Tyrosine Kinase Src Antagonists

The acknowledgement of tyrosine kinase receptors (TKR) and their role in cell signal transduction, particularly regarding cell growth and oncogenesis, has resulted in the development of antibodies and small inhibitory molecules targeting these proteins. In particular, the association of rapid bone growth to OS pathogenesis and the recognition of TKR role in the TGF cell signaling pathway have led to the investigation of agents targeting these receptors [32].

Dasatinib and saracatinib are two small molecules targeting tyrosine kinase Src in investigation. Dasatinib is being investigated in a phase I/II trial of young patients with malignant, metastatic, or recurrent solid tumors (including OS) for assessment of the drug’s side effects and best dose when administered in combination with carboplatin, ifosfamide, and etoposide [30] (Table 1). Results of the role of saracatinib (AZD0530), a selective inhibitor of the Abl and Src kinases, in patients with lung metastasis of OS, have been recently reported in another phase II trial (Table 1). Although saracatinib was well tolerated and demonstrated potential clinical benefit in this patient population, there was no apparent impact on overall survival, and Src inhibition alone may not be sufficient to suppress metastatic progression in OS [37].

### 3.6. Vascular Endothelial Growth Factor (VEGF) Inhibitors

In OS, elevated levels of circulating VEGF produced by the tumor have been associated with increased local microvascular density, development of metastases, and worse prognosis [38].

Bevacizumab has been used to treat high-grade OS, appearing to be highly effective when administered in combination with liposomal doxorubicin [38]. More recently, this monoclonal antibody was introduced in a phase III clinical trial for high-grade OS or malignant fibrous bone histiocytoma in association with ChT. It can be speculated that, given the complexity of angiogenesis, inhibition of a single element in this process (in this case, VEGF) may be insufficient to achieve significant clinical control. It is therefore important to develop new angiogenesis inhibitors with broader specificity [30]. Endostar, an angiogenesis-inhibitor recombinant human endostatin, is currently being investigated in phase II clinical trials of melanoma, colorectal carcinoma, small-cell lung cancer, and high-grade OS. The OS study will be carried out in patients with high-grade non-metastatic disease (Table 1) using endostar in combination with cisplatin, doxorubicin, high-dose methotrexate, and ifosfamide [30].

### 3.7. Mammalian Target of Rapamycin (MTOR) Inhibitors

The mTOR pathway is fulcral not only for mesenchymal stem cells, but also for bone biology, often being regarded as an attractive therapeutic target in OS [30]. mTOR inhibitors suppress OS cell growth in vivo and in vitro and is already being studied in phase II studies (Table 1) [39,40,41,42]. Studies have reported that mTOR inhibitors achieve an increasing anti-tumor effect when combined with other drugs such as anti-osteoporotic drugs, extra-terminal domain protein inhibitors, and conventional ChT drugs [39]. In addition, dual PI3K/mTOR inhibition has shown promising results in treating OS (Table 1) and this anti-tumor activity can be enhanced by MEK/Erk inhibition [39,43,44].

It has been proposed that rapamycin induces apoptosis of OS cells [45] and that treating OS by targeting the mTOR pathway alone could inhibit proliferation and promotion of these tumor cells. However, the pro-apoptotic effect triggers autophagy as an escape pathway, counteracting the anti-tumor effect of mTOR inhibitors and contributing to resistance to these agents. This shows how further investigation is necessary on this subject.

Overall, the use of mTOR inhibitors in association with other drugs may offer a new therapeutic strategy against OS. Nevertheless, the combination of anti-insulin growth factor type 1 receptor antibody and mTOR inhibitor did not show objective results in a phase II trial [46]. In fact, the mTOR signaling pathway seems to be complex in OS, and further studies are needed to develop a combination ChT regimen against OS.

### 3.8. Micro RNA (MiRNA) Therapeutic Targets

MiRNA are small non-coding RNA molecules with gene regulatory functions, namely in post-transcriptional gene expression, resulting in translational inhibition or degradation of the target mRNA [47,48]. These miRNAs have been shown to have a role in cancer biology as their regulatory function seems to affect an important number of downstream genes in cancer [49]. Although most available studies so far are preclinical, these molecules are drawing attention as therapeutic targets as miRNA molecules seem to have an active role in several aspects of OS biology including pathogenesis, metastases development, diagnosis, response and resistance to therapy, and prognosis [50].

miRNAs regulate tumor progression in OS through dysregulation and activation of the Notch and Wnt/β-catenin pathways. miR-26a blocks the Jagged 1/Notch signaling pathway, and Notch2, a key receptor in this pathway that promotes OS cell proliferation, migration, and invasion, is repressed by miR-1296-5p [51,52]. Two other miRNA molecules—miR-34 and miR-200—seem to have an oncosupressor function through Notch1 downregulation and their levels are low in OS [53]. miR-377, miR-425-5p, miR-758, and miR-873 promote OS cell apoptosis through Wnt pathway suppression [54,55,56,57]. Another molecule, miR-940, has a stimulatory role of the Wnt pathway and has been shown to be overexpressed in OS [58]. miR-199a-3p, miR-99a, and miR-140 participate in the PI3K-Akt-mTOR pathway, which is frequently activated in OS [59,60,61].

Several miRNA molecules have been assessed in preclinical studies investigating OS metastases development. Some have been shown to have a protective role, preventing metastases development such as miR-491 and miR-223-3p, while others promote lung metastases such as miR-19a [62,63,64]. Expression levels of some miRNAs may be related to prognosis, not only regarding metastatic status, but also tumor stage and disease outcomes, as in the case of miR-9 and miR-195 [65,66].

Other mechanisms contributing to OS development and progression in which miRNA molecules are involved include microenvironment and extracellular matrix targeting [67], angiogenesis through dysregulation of VEGF ligands and receptors [68] and IL-6 receptor [69], and TGF-β signaling [70].

miRNA expression is also associated with response to treatment and different ChT resistance mechanisms. For example, through inhibition of the cell cycle-regulated nuclear and centrosome protein DTL, miR-215a leads to resistance to methotrexate, a common drug in OS ChT regimens [71].

Overall, by providing multiple therapeutic targets, miRNAs are relevant in OS treatment. However, clinical studies with drugs targeting miRNA are still lacking in OS. To date, only a few studies with these molecules have been conducted in other cancers, mostly basket trials targeting different cancer types, but studies in sarcoma, a rare tumor, and in OS, an even rarer subtype, are scarce.

### 3.9. Signaling Pathways and Epigenetic Regulators

#### 3.9.1. Hedgehog Pathway

The hedgehog (Hh) signaling pathway is a highly evolutionarily conserved pathway that regulates embryonic development, tissue differentiation, and cell growth [72]. As a pathway related to organ development and growth, it is dormant in most adult tissues. However, the Hh pathway is abnormally activated in several cancer types and its role in cancer development including carcinogenesis, invasion, and metastases is well established [73]. The Hh signaling pathway is regulated by many different agents such as proteins (kinases, transcriptional factors, glycoproteins, and pro/anti-apoptotic factors) and noncoding RNA (such as miRNA) [74]. Activation of this pathway by one of three homologous Hh ligands—Sonic Hh, Desert Hh, and Indian Hh—interacting with the Patched (Ptch) 1 transmembrane protein leads to release of Smoothened (Smo), a G protein-coupled receptor, and subsequent activation of the downstream signaling cascade. The Gli family of transcriptional factors (1,2 and 3) is translocated to the nucleus and leads to target gene transcription and Wnt and Noggin protein modulation [74,75]. The three homologous Hh ligands and Ptch can be combined to perform different biological functions during different stages of cancer progression [74].

High levels of Hh signaling are associated with high-grade human OS progression [76]. The gene encoding ribosomal protein S3 (RPS3) is one of the Gli2 transcriptional factor targets. It is overexpressed in patients with OS tumors with lung metastases compared to patients without distant disease, thus making this protein a potential biomarker and therapeutic target for aggressive OS [77]. Yes-associated protein 1 (Yap1), a potent oncogene abnormally overexpressed in various cancers, is regulated by the Hh signaling pathway. This protein is present in high levels in OS tissue. Suppression of this cascade leads to lower levels of Yap1 and its knock-down leads to OS progression inhibition, suggesting that the Hh pathway and Yap1 protein are potential therapeutic targets, along with long-non-coding RNA (lncRNA) H19, which is also abnormally overexpressed in OS tissues [78].

Some target agents with therapeutic potential have been studied. Saridegib, a small-molecule inhibitor of Smo, inhibits Hh signaling pathway in OS [79]. Degalactotigonin (DGT) inhibits the Hh/Gli1 pathway, leading to suppression of cell growth and migration, inhibition of metastases development, and increased apoptosis in OS [80].

#### 3.9.2. Wnt/β-catenin

Wnt/β-catenin signaling is a conserved pathway involved in embryonic development and adult tissue homeostasis through regulation of cell differentiation and proliferation. The interaction of Wnt with Frizzled, and LRP5 or LRP6, activates a molecular cascade, leading to glycogen synthase kinase 3 (GSK3) inhibition. GSK3 inactivates β-catenin by phosphorylation and targeting of proteosomal degradation, meaning that with Wnt activation, β-catenin enters the nucleus to regulate the transcription of target genes [81]. The role of this signaling pathway has been studied and established in several cancers, namely in tumor progression, chemoresistance, and relapse [82]. In OS, the canonical Wnt signaling pathway is abnormally activated, resulting in tumor growth and metastatic spread [83,84]. Although there are a few clinical studies with Wnt signaling inhibitors, studies in OS are preclinical at this point. MiRNAs can also play a role in Wnt/β-catenin inhibition, as shown by suppression of OS progression by miR-429 through targeting of the HOXA9 via Wnt/β-catenin signaling pathway [85]. In vitro and in vivo studies have shown that alantolactone (ALT), a natural eucalyptone sesquiterpene lactone, inhibits cell proliferation, migration, and invasion, promotes apoptosis, arrests cell cycle at G2/M phase, and restrains tumor growth and metastases development in OS. ALT inhibits the activity of Wnt/β-catenin and p38 and of the ERK1/2 and JNK Mitogen Activated Protein Kinases (MAPKs) signaling pathway [86]. Ginsenoside Rg3 inhibits proliferation, migration, and invasion of OS cells by downregulating MMP2, MMP7, and MMP9 expression and suppressing epithelial-mesenchymal transition (EMT) and the Wnt/β-catenin pathway [87].

#### 3.9.3. Notch Signaling Pathway

Notch signaling is another conserved pathway involved in proliferation, apoptosis, migration, and angiogenesis. A cell surface ligand of the Delta–Serrate–Lag family binds to the membrane-bound Notch receptor (Notch1–4) in another cell, leading to the proteolytic cleavage of the receptor by ADAM10 or ADAM 17 and by the γ-secretase complex. This event releases the Notch intracellular domain, which when activated, enters the nucleus and regulates the transcription of several transcription factors involved in progenitor cell survival [88]. Notch1 signaling seems to be activated and dysregulated in OS, promoting tumor invasion and metastases development by way of abnormal differentiation or undifferentiation, leading cells toward malignant transformation by regulating cancer stem cells [89,90].

Preclinical studies have shown that Notch1 may be related to chemoresistance, as in vitro regulation of Notch1 (activation/inhibition) altered cisplatin-induced apoptosis, probably through the activity of the Caspase family of proteases [89]. DAPT, a γ-secretase inhibitor, enhances the sensitivity of resistant OS cells to cisplatin by downregulating Notch signaling [90]. Another γ-secretase inhibitor and CBF1 siRNA slowed OS growth in xenograft models by cell-cycle arrest in G1 [91].

## 4. Immunotherapy

The immune landscape of OS provides several opportunities of immune modulation against neoplastic clones. Immunotherapy approaches for this type of sarcoma can be subdivided in non-specific (immune modulation, cytokine enrichment, immune checkpoint inhibition) and specific (dendritic cell vaccination, chimeric antigen receptor [CAR]-T cells) (Table 1).

### 4.1. Nonspecific Immunotherapy

#### 4.1.1. Cytokines

In the immunoediting process in OS, multiple cells and cytokines appear to play crucial roles in each of the elimination, equilibrium, and escape phases, constituting potential therapeutic targets. During the elimination phase, cells of the innate and adaptive immune system work to detect and destroy tumor cells including CD4+ T-helper (Th) cells, CD8+ cytotoxic T cells, γδ T cells, natural killer (NK) cells, NK T cells, M1 macrophages, and dendritic cells (DC). Additionally, cytokines (such as IL-1, IL-2, IL-6, IL-8, IL-12, IL-18, INF-α, INF-β, INF-γ, and tumor necrosis factor [TNF]-α), growth factors (such as granulocyte-macrophage colony stimulating factor [GM-CSF]), cytolytic proteins (such as perforin), and serine proteases (such as granzyme B) constitute possible targets for anti-cancer immune modulation [92]. Throughout the equilibrium phase, tumor development and growth are controlled by the immune system [92]. In the escape phase, the steadiness between tumor growth and its control by an appropriate immune response fluctuates toward tumor growth. Immune cells conferring tumor tolerance include myeloid-derived suppressor cells, regulatory T cells (Treg), Th17 cells, and M2 macrophages; different immune checkpoint molecules (such as programmed cell death protein 1 [PD-1], cytotoxic T-lymphocyte antigen 4 [CTLA-4], and TIM3), along with other molecules and cytokines (such as LAG3, CD137, MHC class II, FASLG, IL-10, IL-23, TGF-β, INF-γ, scavenger receptor A, arginase, and CD40) may also be susceptible targets for anti-cancer immune modulation [92].

Mifamurtide, a synthetic lipophilic analogue of muramyl dipeptide (the minimal peptidoglycan motif common to Gram-negative and Gram-positive bacteria that can activate the innate immune system), is capable of activating monocytes and macrophages, and subsequently increasing serum levels of TNF-α, IL-1α, IL-1β, IL-6, and IL-8 (cytokines with important roles in bone microenvironment), with engagement of other immune cells [92]. A randomized clinical trial in OS patients showed that the addition of the macrophage-activating agent mifamurtide to a standard ChT regimen resulted in a significant improvement in 6-year overall survival [93]. A supplementary average of 2.58 years of life and 2.20 quality-adjusted life years was verified on patients receiving adjuvant mifarmurtide when compared with patients receiving ChT alone [92]. The SARCOMA13 clinical trial (Table 1) provided evidence regarding the association of mifamurtide and conventional ChT in the treatment of OS patients after surgery [93].

Tumor-infiltrating macrophages coordinate important processes of OS stromal signaling and OS progression, with infiltration of M2-like-tumor-associated-macrophages being linked with OS metastasis and poor disease prognosis [NR]. Strategies that regulate tumor associated macrophages (TAM)-polarization from an M2 phenotype to an M1 phenotype may have beneficial effects on macrophage dependent tumor progression [NR]. Not only mifamurtide (by promoting a TAM polarization towards an intermediate M1/M2 phenotype), but also all trans retinoic acid, esculetin, zoledronate, natalizumab, nivolumab, and pembrolizumab may have the capacity of shaping TAM polarization and their function [NR]. Recent evidence also suggests that blocking PI 3-kinase γ, ERK5-MAPK, and c-Maf pathways (using, for example, nanoparticles loaded with its specific inhibitors) may promote repolarization of TAM to an M1 phenotype with subsequent antitumor and anti-metastatic effects [94]. Finally, the use of chimeric antigen receptor macrophages may also have positive effects on TAM polarization [94] [NR].

#### 4.1.2. Immunomodulating Agents

INF-α and IFN-β have antitumor activity in a variety of malignancies [92]. In OS, specifically, INF-α inhibits cancer clones in vitro and enhances OS sensitivity to chemotherapeutic drugs as doxorubicin, despite showing conflicting evidence regarding survival improvement and tumor regression (in patients with metastatic OS) in clinical trials [92]. A small clinical trial showed that high IL-2 doses were able to induce complete responses in patients with metastatic OS, but at the cost of major toxicities [92]. IL-12 has the ability to inhibit OS growth by upregulating CD95 receptor expression, which reduces the capability of cancer clones to evade immune surveillance [92]. Inhaled GM-CSF induces differentiation and apoptosis in human OS cell lines in vitro, but in clinical studies, it was not associated with improved outcomes and significant immunomodulatory effects in relapsing OS patients with pulmonary metastases [92]. Inhaled GM-CSF is also being investigated in OS patients with pulmonary recurrence [95].

IFN-α has been used in OS as unique surgery adjunct for two decades, with apparent clinical efficacy. The EURAMOS-1 study (Table 1) aimed to investigate whether the addition of pegylated IFNα-2b as maintenance therapy after postoperative ChT with doxorubicin, cisplatin, and methotrexate improved event-free survival and overall survival in patients with resectable OS and good histological response at 10 weeks preoperatively [96].

#### 4.1.3. Immune Checkpoint Inhibitors

As previously mentioned, the high levels of genomic instability with potentially associated high mutational load in OS have raised interest in targeting immune checkpoint pathways in this tumor. Additionally, pre-clinical data highlighted the putative specific relevance of immune checkpoint inhibitors (ICI) for OS: CTLA-4 polymorphisms are associated with higher risk of developing OS [92]; the combination of tumor lysate-pulsated dendritic cells with an antibody against CTLA-4 decreases immunosuppressive Treg and increases cytotoxic T cells in a murine model of metastatic OS, with associated survival gain [92,97]; PD-1 and programmed cell death ligand 1 (PD-L1) expression is increased in OS patients and correlated with poor prognosis [97]; PD-1 inhibition results in anti-metastatic effects in OS murine models [97]; and PD-1 blockade in a murine model of metastatic OS (with documented lung metastases) decreases the number of OS lung nodules by increasing the macrophage tumor infiltration and polarization from the M2 to M1 phenotype [97]. However, results from clinical trials (Table 1) testing single-approach immune checkpoint inhibition have been disappointing. One trial with the anti-PD-L1 avelumab is currently recruiting and three trials using the anti-PD-L1 pembrolizumab and nivolumab showed poor results (only one—5%—of 22 patients showed partial response with pembrolizumab [97]) and stopped recruitment or were suspended due to risk of immune-related side effects [93]. Hence, the discovery of ICIs that show therapeutic efficacy in OS is still an unmet need. OS are typically considered “cold tumors”, given the low inflammatory immune cell infiltration, which is a plausible explanation for the observed resistance to immune checkpoint blockade. A reasonable strategy would be to convert OS into “hot tumors” (with enhanced infiltration of inflammatory immune cells), maximizing the potential benefit of ICIs. Combining CTLA-4 and PD-1, ICIs with tumor infiltrating lymphocytes, anti-PD-1 with low-dose cyclophosphamide, ICIs with CAR-T cells or bispecific T-cell engager antibodies, or ICIs with dendritic cell vaccines are possibilities for increasing the efficacy of immune-mediated anti-tumor response through immune modulation of different pathways and at different levels [98].

### 4.2. Tumor Specific Immunotherapy

#### 4.2.1. Dendritic Cell Peptide Vaccines

Dendritic cells (DC) are professional antigen-presenting cells (APC) with the capacity of taking up and presenting neoplastic antigens to naïve T cells, promoting their differentiation into tumor killers [97]. OS, like a wide variety of malignant neoplasms, has the ability to reduce antigen presentation by APCs, resulting in immunosuppression with blunted anti-cancer immune responses [97]. DCs can be isolated from peripheral blood mononuclear cells, matured, and loaded ex vivo with tumor antigens with defined mixtures and infused back into the patient, bypassing the above-mentioned mechanism of immunosuppression and boosting the anti-cancer immune response [97]. DC vaccines can be co-cultured with peptides, proteins, or tumor-cell lysates, transfected with DNA, RNA coding for antigens, or total RNA derived from tumor cells or fused with devitalized tumor cells [97]. DC vaccines, alone or in combination with other targeted drugs (like anti-TGF-β antibodies), have been shown to promote primary and metastatic tumor growth inhibition and remodeling of tumor microenvironment with reduced Tregs, reduced immunosuppressive cytokines, and increased CD8+ T lymphocytes in OS in different studies [97]. Aside from these studies, the benefits of these vaccines in OS treatment were less pronounced in clinical trials, with only two out of 12 patients exhibiting an anti-tumor immune response and none exhibiting clinical effects [97]. The compromised quantity and quality of immune effector cells in OS patients (normally already submitted to a ChT course that negatively affects this cell population), poor migration of effector cells to tumor site (related with down-regulation of chemokine expression), and presence of other immunosuppressive mechanisms (like immune checkpoints, highlighting the previously mentioned importance of combining approaches) may explain this lack of clinical benefit [97]. DC vaccines tested in OS were well tolerated and safe in clinical trials [97,98].

#### 4.2.2. CAR-T Cells

Adoptive cell therapy has the goal of supplying patients with cytolytic cells, amplifying the magnitude of anti-tumor response [98]. T cells, in particular, can be edited to respond with high affinity to specific antigens without the need for peptide recognition in the context of HLA presentation [98]. These cells are designated CAR-T cells and comprise an extracellular domain derived from a monoclonal antibody specific to a tumor surface antigen, a spacer, and a transmembrane and intracellular domain [98]. The clinical success of different adoptive therapies, mainly relying on the use of CAR-T cells, in B-cell malignancies led to multiple attempts to apply this strategy in solid tumors [93]. OS-specific antigens have been difficult to identify, as mesenchymal cells lack specific markers and tend to be non-immunogenic [92]. Several antigens expressed in OS are also found in healthy tissues [92]. The human epidermal growth factor-2 (HER-2) is expressed in OS at low levels and may be susceptible to HER-2 CAR-T cell targeting [92]; disialyl ganglioside (GD-2) is expressed in half of OSs and may also represent a treatment target [92]; IGF receptor-1 (IGFR1), tyrosine orphan-like receptor-1 (ROR1), folate receptor-α, and CD146 are also potential targets [92]. HER-2 CAR-T cells have been shown to promote tumor regression in animal models and to induce no toxicity, promote disease stability, and, given the 6-week stability of these cells, have a long-lasting effect in a phase I/II clinical trial in patients with HER-2 positive sarcoma [93]. IGFR1 and ROR1 CAR-T cells derived from a sarcoma patient significantly suppressed tumor growth in both localized and disseminated sarcoma xenograft models [93]. The main issue with the use of CAR-T cells in OS is target access through the osteoid bone tumor matrix, but OS treatment with CAR-T cells is still being investigated [93].

Improved knowledge and better understanding of the crosstalk between OS cells, osteoclasts, and immune cells may lead to the optimization of immunotherapy use in OS [99]. This therapeutic approach will predictably have a more prominent role in the adjuvant setting and greatest efficacy in micrometastatic disease [92]. Combining immunotherapy with other types of anti-cancer therapy like ChT and combining different immunotherapy modalities will predictably maximize the efficacy of this therapeutic intervention.

## 5. Conclusions

The burden of OS to patients and the community is currently high, as available treatments combine ChT, often disabling surgery and prolonged rehabilitation periods. New targets are necessary to improve the survival and quality of life for these patients. OS subtypes are characterized by a large number of genomic alterations and mutations, which often provide great potential for target therapies. Several trials are ongoing in order to achieve this goal. In the palliative setting, cytotoxic treatments in monotherapy are still the standard, but cytotoxic treatments are also being studied in combination with target therapy as mentioned previously in this paper. The therapeutic combination may allow a synergistic or additive effect and, therefore, it may be possible to reduce the doses of conventional chemotherapy, decreasing toxicity. However, clinical research is still limited, and several drugs have failed to show clinical benefit.

## Figures and Tables

**Table 1:** Clinical trials with potential new molecular targets.

Molecular Target	Research Drug	Clinical Trial	Results and Conclusions
IGF-1 receptor antagonists	Figitumumab (CP-751.871)	Phase I/II trial NCT00560235	Figitumumab had modest activity as single-agent in advanced Ewing sarcoma
	Cixutumumab (BMI A12)	Phase II trials NCT00831844	Cixutumumab was well tolerated in children with refractory solid tumors, with limited objective single-agent activity and prolonged stable disease in 15% of patients
		NCT01016015; NCT01614795	The combination of cixutumumab and temsirolimus showed clinical activity in patients with sarcoma, but no objective responses in a phase II trial of pediatric and young adults with recurrent or refractory sarcoma
	Robatumumab (SCH717454)	Phase II trial—NCT00617890	Additional research is needed to identify responders, with low disease burden as an important factor for osteosarcoma response
HER2/neu receptor blockers	Trastuzumab	Cooperative Children’s Oncology Group (COG) phase II study	Trastuzumab can be safely delivered in combination with anthracycline-based ChT and dexrazoxane, but its therapeutic benefit remains uncertain
PDFG inhibitors	Imatinib	Cooperative Children’s Oncology Group (COG) phase II study	Imatinib failed to demonstrate activity against OS as single agent at conventional doses
RANKL and bisphosphonate inhibitors	Pamidronate	Phase II trial	Pamidronate can be safely incorporated into ChT regimens for the treatment of OS without impairing its effectiveness and can also improve the durability of limb reconstruction. Survival results remain to be determined
Tyrosine kinase Src antagonists	Dasatinib	Phase I trial—NCT00316953	Drug disposition and tolerability of dasatinib in children were similar to those observed in adult patients
	Saracatinib	Phase II trial—NCT00559507	Saracatinib was well tolerated, with a suggestion of potential clinical benefit, but no apparent impact in survival
VEGF inhibitors	Bevacizumab	Phase II trial—NCT0066734; NCT00667342	The estimated 4-year event-free survival (EFS) rate and overall survival rate were 57.5 6 10.0% and 83.4 6 7.8%, respectively. Eight (28%) of 29 evaluable patients had good histologic response (<5% viable tumor) to preoperative chemo- therapy. The addition of bevacizumab to MAP for localized osteosarcoma is feasible but frequent wound complications are encountered. The observed histologic response and EFS do not support further evaluation of bevacizumab in osteosarcoma
	Endostar	Phase II trial—NCT01002092	The study’s primary endpoint is safety and efficacy of Endostar combined with ChT in non-metastatic OS patients—trial without published results
mTOR inhibitors	Sirolimus	Phase II trial—NCT02429973	Gemcitabine plus sirolimus exhibited satisfactory antitumor activity and safety in this OS population, exceeding the prespecified 40% of 4-month PFS
	Ridaforolimus	Phase II NCT00112372 NCT00093080	OS patient had a PR
	Tensirolimus	Discussed in the IGF-1 receptor antagonist section	
Cytocines	Mifamurtide in combination with postoperative ChT	Phase II Trial (SARCOMA13) NCT03643133	Improvement in 6-year overall survival and an additional average of 2.58 years of life and 2.20 quality-adjusted life years vs. ChT alone.
Immunomodulating agents	INFα-2b in combination with postoperative doxorubicin, cisplatin and methotrexate	Phase III Trial (EURAMOS-1) NCT00134030	Improvement in event-free and overall survival in resectable OS, with good histological response after preoperatively ChT
Immune checkpoint inhibitors	Pembrolizumab	Phase II Trials Pembrolizumab (NCT02301039 and NCT03013127) Avelumab (NCT03006848) Nivolumab (NCT02304458	Disappointing results (5% of patients with PR to pembrolizumab); only avelumab trial currently recruiting; other trials suspended due to immune side effects
	Avelumab		
	Nivolumab		
Dendritic cell peptide vaccines	DC vaccines + decitabine or gemcitabine pretreatment	Phase I/II studies DC vaccine + decitabine (NCT01241162) DC vaccine + gemcitabine (NCT01803152)	Primary and metastatic tumor growth inhibition and remodeling of tumor microenvironment with reduced Treg and immunosuppressive cytokines and increased CD8+ T lymphocytes, with small outcome benefits in clinical trials
CAR-T cells	HER-2 CARt Cells IGFR1 CARt Cells ROR1 CARt Cells	Phase I/II Trials	HER-2 CAR-T cells: Tumor regression in animal models with no toxicity, disease stability and, given the 6-week stability of these cells, long-lasting effect in a phase I/II trial in HER-2 positive sarcoma. IGFR1/ROR1 CAR-T cells: Suppressed tumor growth in both localized and disseminated sarcoma xenograft models

CAR, chimeric antigen receptor; ChT, chemotherapy; DC, dendritic cells; HER, human epidermal growth factor receptor; IGFR, insulin growth factor receptor; OS, osteosarcoma; PDGF, platelet-derived growth factor; PFS, progression free survival; PR, partial response.

## Abbreviations

APC	Antigen-presenting cells
CAR-T	Chimeric antigen receptor T
ChT	Chemotherapy
COG	Children’s Oncology Group
DC	Dendritic cells
GM-CSF	Granulocyte-macrophage colony stimulating factor
HER2	Human Epidermal growth factor Receptor 2
Hh	Hedgehog
ICI	Immune checkpoint inhibitors
IGF	Insulin-like growth factor
IGF-1R	IGF-1 receptor
IL	Interleukin
INF	Interferon
miRNA	microRNA
mTOR	Mammalian target of rapamycin
NK	Natural killer
OS	Osteosarcoma
PD-L1	Programmed cell death ligand 1
PD-1	Programmed cell death protein 1
PDGF	Platelet-derived growth factor
PDGFR	Platelet-derived growth factor receptor
PD-L1	Programmed cell death ligand 1
Ptch	Patched
PTHrP	Parathyroid hormone- related peptide
RANKL	Receptor activator of nuclear factor kappa-Β ligand
Smo	Smoothened
TGF	Transforming growth factor
Th	T-helper
TNF	Tumor necrosis factor
Treg	Regulatory T cells
uPA	Urokinase plasminogen activator
VEGF	Vascular endothelial growth factor

## References

[1] Broadhead M.L., Clark J.C.M., Myers D.E., Dass C.R., Choong P.F.M. (2011). The Molecular Pathogenesis of Osteosarcoma: A Review. Sarcoma.

[2] Isakoff M.S., Bielack S.S., Meltzer P., Gorlick R. (2015). Osteosarcoma: Current Treatment and a Collaborative Pathway to Success. J. Clin. Oncol. Off. J. Am. Soc. Clin. Oncol..

[3] Lindsey B.A., Markel J.E., Kleinerman E.S. (2017). Osteosarcoma Overview. Rheumatol.

[4] Sadykova L.R., Ntekim A.I., Muyangwa-Semenova M., Rutland C.S., Jeyapalan J.N., Blatt N., Rizvanov A.A. (2020). Epidemiology and Risk Factors of Osteosarcoma. Cancer Investig..

[5] Petrilli A.S., de Camargo B., Filho V.O., Bruniera P., Brunetto A.L., Jesus-Garcia R., Camargo O.P., Pena W., Péricles P., Davi A. (2006). Results of the Brazilian Osteosarcoma Treatment Group Studies III and IV: Prognostic factors and impact on survival. J. Clin. Oncol. Off. J. Am. Soc. Clin. Oncol..

[6] Tunn P.U., Reichardt P. (2007). Chemotherapy for osteosarcoma without high-dose methotrexate: A 12-year follow-up on 53 patients. Onkologie.

[7] Bielack S.S., Kempf-Bielack B., Delling G., Exner G.U., Flege S., Helmke K., Kotz R., Salzer-Kuntschik M., Werner M., Winkelmann W. (2002). Prognostic factors in high-grade osteosarcoma of the extremities or trunk: An analysis of 1,702 patients treated on neoadjuvant cooperative osteosarcoma study group protocols. J. Clin. Oncol. Off. J. Am. Soc. Clin. Oncol..

[8] Meyers P.A., Gorlick R., Heller G., Casper E., Lane J., Huvos A.G., Healey J.H. (1998). Intensification of preoperative chemotherapy for osteogenic sarcoma: Results of the Memorial Sloan-Kettering (T12) protocol. J. Clin. Oncol. Off. J. Am. Soc. Clin. Oncol..

[9] Whelan J.S., Bielack S.S., Marina N., Smeland S., Jovic G., Hook J.M., Krailo M., Anninga J., Butterfass-Bahloul T., Böhling T. (2015). EURAMOS-1, an international randomised study for osteosarcoma: Results from pre-randomisation treatment. Ann. Oncol. Off. J. Eur. Soc. Med Oncol..

[10] Yang Y., Han L., He Z., Li X., Yang S., Yang J., Zhang Y., Li D., Yang Y., Yang Z. (2017). Advances in limb salvage treatment of osteosarcoma. J. Bone Oncol..

[11] Chou A.J., Gorlick R. (2006). Chemotherapy resistance in osteosarcoma: Current challenges and future directions. Expert Rev. Anticancer.

[12] Abarrategi A., Tornin J., Martinez-Cruzado L., Hamilton A., Martinez-Campos E., Rodrigo J.P., González M.V., Baldini N., Garcia-Castro J., Rodriguez R. (2016). Osteosarcoma: Cells-of-Origin, Cancer Stem Cells, and Targeted Therapies. Stem Cells Int..

[13] Ta H.T., Dass C.R., Choong P.F., Dunstan D.E. (2009). Osteosarcoma treatment: State of the art. Cancer Metastasis. Rev..

[14] Marina N., Gebhardt M., Teot L., Gorlick R. (2004). Biology and therapeutic advances for pediatric osteosarcoma. Oncologist.

[15] Gamberi G., Benassi M.S., Bohling T., Ragazzini P., Molendini L., Sollazzo M.R., Pompetti F., Merli M., Magagnoli G., Balladelli A. (1998). C-myc and c-fos in human osteosarcoma: Prognostic value of mRNA and protein expression. Oncology.

[16] Shimizu T., Ishikawa T., Sugihara E., Kuninaka S., Miyamoto T., Mabuchi Y., Matsuzaki Y., Tsunoda T., Miya F., Morioka H. (2010). c-MYC overexpression with loss of Ink4a/Arf transforms bone marrow stromal cells into osteosarcoma accompanied by loss of adipogenesis. Oncogene.

[17] Franchi A., Arganini L., Baroni G., Calzolari A., Capanna R., Campanacci D., Caldora P., Masi L., Brandi M.L., Zampi G. (1998). Expression of transforming growth factor β isoforms in osteosarcoma variants: Association of tgfβ1 with high-grade osteosarcomas. J. Pathol..

[18] Rikhof B., de Jong S., Suurmeijer A.J.H., Meijer C., van der Graaf W.T.A. (2009). The insulin-like growth factor system and sarcomas. J. Pathol..

[19] Gagiannis S., Müller M., Uhlemann S., Koch A., Melino G., Krammer P.H., Nawroth P.P., Brune M., Schilling T. (2009). Parathyroid hormone-related protein confers chemoresistance by blocking apoptosis signaling via death receptors and mitochondria. Int. J. Cancer.

[20] Jan Y., Matter M., Pai J.-T., Chen Y.-L., Pilch J., Komatsu M., Ong E., Fukuda M., Ruoslahti E. (2004). A Mitochondrial Protein, Bit1, Mediates Apoptosis Regulated by Integrins and Groucho/TLE Corepressors. Cell.

[21] Janes S.M., Watt F.M. (2004). Switch from alphavbeta5 to alphavbeta6 integrin expression protects squamous cell carcinomas from anoikis. J Cell Biol.

[22] Shibuya M., Claesson-Welsh L. (2006). Signal transduction by VEGF receptors in regulation of angiogenesis and lymphangiogenesis. Exp. Cell Res..

[23] Matsumoto T., Mugishima H. (2006). Signal transduction via vascular endothelial growth factor (VEGF) receptors and their roles in atherogenesis. J. Atheroscler. Thromb..

[24] Yancopoulos G.D., Davis S., Gale N.W., Rudge J.S., Wiegand S.J., Holash J. (2000). Vascular-specific growth factors and blood vessel formation. Nature.

[25] Lobov I.B., Renard R.A., Papadopoulos N., Gale N.W., Thurston G., Yancopoulos G.D., Wiegand S.J. (2007). Delta-like ligand 4 (Dll4) is induced by VEGF as a negative regulator of angiogenic sprouting. Proc. Natl. Acad. Sci. USA.

[26] Masson V., De La Ballina L.R., Munaut C., Wielockx B., Jost M., Maillard C., Blacher S., Bajou K., Itoh T., Itohara S. (2005). Contribution of host MMP-2 and MMP-9 to promote tumor vascularization and invasion of malignant keratinocytes. FASEB J..

[27] Hofbauer L.C., Heufelder A.E. (1998). Osteoprotegerin and its cognate ligand: A new paradigm of osteoclastogenesis. Eur. J. Endocrinol..

[28] Lamoureux F., Richard P., Wittrant Y., Battaglia S., Pilet P., Trichet V., Blanchard F., Gouin F., Pitard B., Heymann D. (2007). Therapeutic relevance of osteoprotegerin gene therapy in osteosarcoma: Blockade of the vicious cycle between tumor cell proliferation and bone resorption. Cancer Res..

[29] Wu C.-C., Beird H.C., Andrew Livingston J., Advani S., Mitra A., Cao S., Reuben A., Ingram D., Wang W.-L., Ju Z. (2020). Immuno-genomic landscape of osteosarcoma. Nat. Commun..

[30] Hattinger C.M., Pasello M., Ferrari S., Picci P., Serra M. (2010). Emerging drugs for high-grade osteosarcoma. Expert Opin. Emerg. Drugs.

[31] Scotlandi K., Picci P., Kovar H. (2009). Targeted therapies in bone sarcomas. Curr. Cancer Drug Targets.

[32] Macaulay V.M., Middleton M.R., Eckhardt S.G., Rudin C.M., Juergens R.A., Gedrich R., Gogov S., McCarthy S., Poondru S., Stephens A.W. (2016). Phase I Dose-Escalation Study of Linsitinib (OSI-906) and Erlotinib in Patients with Advanced Solid Tumors. Clin. Cancer Res. Off. J. Am. Assoc. Cancer Res..

[33] Anderson P.M., Bielack S.S., Gorlick R.G., Skubitz K., Daw N.C., Herzog C.E., Monge O.R., Lassaletta A., Boldrini E., Pápai Z. (2016). A phase II study of clinical activity of SCH 717454 (robatumumab) in patients with relapsed osteosarcoma and Ewing sarcoma. Pediatric Blood Cancer.

[34] Ebb D., Meyers P., Grier H., Bernstein M., Gorlick R., Lipshultz S.E., Krailo M., Devidas M., Barkauskas D.A., Siegal G.P. (2012). Phase II trial of trastuzumab in combination with cytotoxic chemotherapy for treatment of metastatic osteosarcoma with human epidermal growth factor receptor 2 overexpression: A report from the children’s oncology group. J. Clin. Oncol. Off. J. Am. Soc. Clin. Oncol..

[35] Kubo T., Piperdi S., Rosenblum J., Antonescu C.R., Chen W., Kim H.S., Huvos A.G., Sowers R., Meyers P.A., Healey J.H. (2008). Platelet-derived growth factor receptor as a prognostic marker and a therapeutic target for imatinib mesylate therapy in osteosarcoma. Cancer.

[36] Meyers P.A., Healey J.H., Chou A.J., Wexler L.H., Merola P.R., Morris C.D., Laquaglia M.P., Kellick M.G., Abramson S.J., Gorlick R. (2011). Addition of pamidronate to chemotherapy for the treatment of osteosarcoma. Cancer.

[37] Baird K., Glod J., Steinberg S.M., Reinke D., Pressey J.G., Mascarenhas L., Federman N., Marina N., Chawla S., Lagmay J.P. (2020). Results of a Randomized, Double-Blinded, Placebo-Controlled, Phase 2.5 Study of Saracatinib (AZD0530), in Patients with Recurrent Osteosarcoma Localized to the Lung. Sarcoma.

[38] Hughes D.P.M. (2009). Novel agents in development for pediatric sarcomas. Curr. Opin. Oncol..

[39] Ding L., Congwei L., Bei Q., Tao Y., Ruiguo W., Heze Y., Bo D., Zhihong L. (2016). mTOR: An attractive therapeutic target for osteosarcoma?. Oncota160rget.

[40] Liu P.Y., Zhang W.B., Wang J., Shen Y.H., Wei Y.Y. (2013). Inhibitory effect and significance of rapamycin on the mammalian target of rapamycin signaling pathway in osteosarcoma stem cells and osteosarcoma cells. Chin. J. Oncol..

[41] Hua Y., Zhang Z., Li J., Li Q., Hu S., Li J., Sun M., Cai Z. (2011). Oleanolic acid derivative Dex-OA has potent anti-tumor and anti-metastatic activity on osteosarcoma cells in vitro and in vivo. Investig. New Drugs.

[42] Chawla S.P., Staddon A.P., Baker L.H., Schuetze S.M., Tolcher A.W., D’Amato G.Z., Blay J.Y., Mita M.M., Sankhala K.K., Berk L. (2012). Phase II study of the mammalian target of rapamycin inhibitor ridaforolimus in patients with advanced bone and soft tissue sarcomas. J. Clin. Oncol. Off. J. Am. Soc. Clin. Oncol..

[43] Gobin B., Battaglia S., Lanel R., Chesneau J., Amiaud J., Rédini F., Ory B., Heymann D. (2014). NVP-BEZ235, a dual PI3K/mTOR inhibitor, inhibits osteosarcoma cell proliferation and tumor development in vivo with an improved survival rate. Cancer Lett..

[44] Zhu Y.R., Min H., Fang J.F., Zhou F., Deng X.W., Zhang Y.Q. (2015). Activity of the novel dual phosphatidylinositol 3-kinase/mammalian target of rapamycin inhibitor NVP-BEZ235 against osteosarcoma. Cancer Biol. Ther..

[45] Grignani G., Palmerini E., Ferraresi V., D’Ambrosio L., Bertulli R., Asaftei S.D., Tamburini A., Pignochino Y., Sangiolo D., Marchesi E. (2015). Sorafenib and everolimus for patients with unresectable high-grade osteosarcoma progressing after standard treatment: A non-randomised phase 2 clinical trial. Lancet. Oncol..

[46] Thompson P.A., Chintagumpala M. (2012). Targeted therapy in bone and soft tissue sarcoma in children and adolescents. Curr. Oncol. Rep..

[47] Bartel D.P. (2004). MicroRNAs: Genomics, biogenesis, mechanism, and function. Cell.

[48] Bartel D.P. (2009). MicroRNAs: Target recognition and regulatory functions. Cell.

[49] Slack F.J., Chinnaiyan A.M. (2019). The Role of Non-coding RNAs in Oncology. Cell.

[50] Lei Y., Junxin C., Yongcan H., Xiaoguang L., Binsheng Y. (2020). Role of microRNAs in the crosstalk between osteosarcoma cells and the tumour microenvironment. J. Bone Oncol..

[51] Lu J., Song G., Tang Q., Yin J., Zou C., Zhao Z., Xie X., Xu H., Huang G., Wang J. (2017). MiR-26a inhibits stem cell-like phenotype and tumor growth of osteosarcoma by targeting Jagged1. Oncogene.

[52] Wang L., Hu K., Chao Y., Wang X. (2020). MicroRNA-1296-5p suppresses the proliferation, migration, and invasion of human osteosarcoma cells by targeting NOTCH2. J. Cell. Biochem..

[53] Li Y., Zhang J., Zhang L., Si M., Yin H., Li J. (2013). Diallyl trisulfide inhibits proliferation, invasion and angiogenesis of osteosarcoma cells by switching on suppressor microRNAs and inactivating of Notch-1 signaling. Carcinogenesis.

[54] Liu Y., Wang Y., Yang H., Zhao L., Song R., Tan H., Wang L. (2019). MicroRNA-873 targets HOXA9 to inhibit the aggressive phenotype of osteosarcoma by deactivating the Wnt/β-catenin pathway. Int. J. Oncol..

[55] Ren J., Yang M., Xu F., Chen J. (2019). microRNA-758 inhibits the malignant phenotype of osteosarcoma cells by directly targeting HMGA1 and deactivating the Wnt/β-catenin pathway. Am. J. Cancer Res..

[56] Xia P., Gu R., Zhang W., Shao L., Li F., Wu C., Sun Y. (2019). MicroRNA-377 exerts a potent suppressive role in osteosarcoma through the involvement of the histone acetyltransferase 1-mediated Wnt axis. J. Cell. Physiol..

[57] Yang G., Zhang C., Wang N., Chen J. (2019). miR-425-5p decreases LncRNA MALAT1 and TUG1 expressions and suppresses tumorigenesis in osteosarcoma via Wnt/β-catenin signaling pathway. Int. J. Biochem. Cell Biol..

[58] Lin Z.-W., Zhang W., Jiang S.-D., Wei W.-B., Li X.-F. (2019). Inhibition of microRNA-940 suppresses the migration and invasion of human osteosarcoma cells through the secreted frizzled-related protein 1–mediated Wnt/β-catenin signaling pathway. J. Cell. Biochem..

[59] Duan Z., Choy E., Harmon D., Liu X., Susa M., Mankin H., Hornicek F. (2011). MicroRNA-199a-3p Is Downregulated in Human Osteosarcoma and Regulates Cell Proliferation and Migration. Mol. Cancer Ther..

[60] Ji X., Wang E., Tian F. (2018). MicroRNA-140 suppresses osteosarcoma tumor growth by enhancing anti-tumor immune response and blocking mTOR signaling. Biochem. Biophys. Res. Commun..

[61] Zhao J., Chen F., Zhou Q., Pan W., Wang X., Xu J., Ni L., Yang H. (2016). Aberrant expression of microRNA-99a and its target gene mTOR associated with malignant progression and poor prognosis in patients with osteosarcoma. Onco-Targets Ther..

[62] Wang S.-N., Luo S., Liu C., Piao Z., Gou W., Wang Y., Guan W., Li Q., Zou H., Yang Z.-Z. (2017). miR-491 Inhibits Osteosarcoma Lung Metastasis and Chemoresistance by Targeting αB-crystallin. Mol. Ther..

[63] Ji Q., Xu X., Song Q., Xu Y., Tai Y., Goodman S.B., Bi W., Xu M., Jiao S., Maloney W.J. (2018). miR-223-3p Inhibits Human Osteosarcoma Metastasis and Progression by Directly Targeting CDH6. Mol. Ther..

[64] Zou Q., Xiao X., Liang Y., Peng L., Guo Z., Li W., Yu W. (2018). miR-19a-mediated downregulation of RhoB inhibits the dephosphorylation of AKT1 and induces osteosarcoma cell metastasis. Cancer Lett..

[65] Cai H., Zhao H., Tang J., Wu H. (2015). Serum miR-195 is a diagnostic and prognostic marker for osteosarcoma. J. Surg. Res..

[66] Fei D., Li Y., Zhao D., Zhao K., Dai L., Gao Z. (2014). Serum miR-9 as a prognostic biomarker in patients with osteosarcoma. J. Int. Med. Res..

[67] Andersen G.B., Knudsen A., Hager H., Hansen L.L., Tost J. (2018). miRNA profiling identifies deregulated miRNAs associated with osteosarcoma development and time to metastasis in two large cohorts. Mol. Oncol..

[68] Zhang L., Lv Z., Xu J., Chen C., Ge Q., Li P., Wei D., Wu Z., Sun X. (2018). MicroRNA-134 inhibits osteosarcoma angiogenesis and proliferation by targeting the VEGFA/VEGFR1 pathway. FEBS J..

[69] Liu S.-Y., Deng S.-Y., He Y.-B., Ni G.-X. (2017). miR-451 inhibits cell growth, migration and angiogenesis in human osteosarcoma via down-regulating IL 6R. Biochem. Biophys. Res. Commun..

[70] Lamora A., Talbot J., Mullard M., Brounais-Le Royer B., Redini F., Verrecchia F. (2016). TGF-β Signaling in Bone Remodeling and Osteosarcoma Progression. J. Clin. Med..

[71] Song B., Wang Y., Titmus M.A., Botchkina G., Formentini A., Kornmann M., Ju J. (2010). Molecular mechanism of chemoresistance by miR-215 in osteosarcoma and colon cancer cells. Mol. Cancer.

[72] Varjosalo M., Taipale J. (2008). Hedgehog: Functions and mechanisms. Genes Dev..

[73] Nwabo Kamdje A.H., Takam Kamga P., Tagne Simo R., Vecchio L., Seke Etet P.F., Muller J.M., Bassi G., Lukong E., Kumar Goel R., Mbo Amvene J. (2017). Developmental pathways associated with cancer metastasis: Notch, Wnt, and Hedgehog. Cancer Biol. Med..

[74] Yao Z., Han L., Chen Y., He F., Sun B., Kamar S., Zhang Y., Yang Y., Wang C., Yang Z. (2018). Hedgehog signalling in the tumourigenesis and metastasis of osteosarcoma, and its potential value in the clinical therapy of osteosarcoma. Cell Death Dis..

[75] Carballo G.B., Honorato J.R., de Lopes G.P.F., Spohr T.C.L.d.S.e. (2018). A highlight on Sonic hedgehog pathway. Cell Commun. Signal..

[76] Lo W.W., Pinnaduwage D., Gokgoz N., Wunder J.S., Andrulis I.L. (2014). Aberrant Hedgehog Signaling and Clinical Outcome in Osteosarcoma. Sarcoma.

[77] Nagao-Kitamoto H., Setoguchi T., Kitamoto S., Nakamura S., Tsuru A., Nagata M., Nagano S., Ishidou Y., Yokouchi M., Kitajima S. (2015). Ribosomal protein S3 regulates GLI2-mediated osteosarcoma invasion. Cancer Lett..

[78] Chan L.H., Wang W., Yeung W., Deng Y., Yuan P., Mak K.K. (2014). Hedgehog signaling induces osteosarcoma development through Yap1 and H19 overexpression. Oncogene.

[79] Lo W.W., Wunder J.S., Dickson B.C., Campbell V., McGovern K., Alman B.A., Andrulis I.L. (2014). Involvement and targeted intervention of dysregulated Hedgehog signaling in osteosarcoma. Cancer.

[80] Zhao Z., Jia Q., Wu M.S., Xie X., Wang Y., Song G., Zou C.Y., Tang Q., Lu J., Huang G. (2018). Degalactotigonin, a Natural Compound from Solanum nigrum L., Inhibits Growth and Metastasis of Osteosarcoma through GSK3β Inactivation-Mediated Repression of the Hedgehog/Gli1 Pathway. Clin. Cancer Res. Off. J. Am. Assoc. Cancer Res..

[81] Niehrs C. (2012). The complex world of WNT receptor signalling. Nat. Rev. Mol. Cell Biol..

[82] Duchartre Y., Kim Y.-M., Kahn M. (2016). The Wnt signaling pathway in cancer. Crit. Rev. Oncol. Hematol..

[83] Chen C., Zhao M., Tian A., Zhang X., Yao Z., Ma X. (2015). Aberrant activation of Wnt/β-catenin signaling drives proliferation of bone sarcoma cells. Oncotarget.

[84] Iwaya K., Ogawa H., Kuroda M., Izumi M., Ishida T., Mukai K. (2003). Cytoplasmic and/or nuclear staining of beta-catenin is associated with lung metastasis. Clin. Exp. Metastasis.

[85] Sun L., Wang L., Luan S., Jiang Y., Wang Q. (2020). miR-429 inhibits osteosarcoma progression by targeting HOXA9 through suppressing Wnt/β-catenin signaling pathway. Oncol. Lett..

[86] Yang C., Zhang L., Huang H., Yuan X., Zhang P., Ye C., Wei M., Huang Y., Luo X., Luo J. (2020). Alantolactone inhibits proliferation, metastasis and promotes apoptosis of human osteosarcoma cells by suppressing Wnt/β-catenin and MAPKs signaling pathways. Genes Dis..

[87] Mao X., Jin Y., Feng T., Wang H., Liu D., Zhou Z., Yan Q., Yang H., Yang J., Yang J. (2020). Ginsenoside Rg3 Inhibits the Growth of Osteosarcoma and Attenuates Metastasis through the Wnt/*β*-Catenin and EMT Signaling Pathway. Evid. Based Complement. Altern. Med..

[88] McManus M.M., Weiss K.R., Hughes D.P. (2014). Understanding the role of Notch in osteosarcoma. Adv. Exp. Med. Biol..

[89] Wang L., Jin F., Qin A., Hao Y., Dong Y., Ge S., Dai K. (2014). Targeting Notch1 signaling pathway positively affects the sensitivity of osteosarcoma to cisplatin by regulating the expression and/or activity of Caspase family. Mol. Cancer.

[90] Dai G., Deng S., Guo W., Yu L., Yang J., Zhou S., Gao T. (2019). Notch pathway inhibition using DAPT, a γ-secretase inhibitor (GSI), enhances the antitumor effect of cisplatin in resistant osteosarcoma. Mol. Carcinog..

[91] Tanaka M., Setoguchi T., Hirotsu M., Gao H., Sasaki H., Matsunoshita Y., Komiya S. (2009). Inhibition of Notch pathway prevents osteosarcoma growth by cell cycle regulation. Br. J. Cancer.

[92] Kansara M., Teng M.W., Smyth M.J., Thomas D.M. (2014). Translational biology of osteosarcoma. Nat. Rev. Cancer.

[93] Corre I., Verrecchia F., Crenn V., Redini F., Trichet V. (2020). The Osteosarcoma Microenvironment: A Complex But Targetable Ecosystem. Cells.

[94] Cersosimo F., Lonardi S., Bernardini G., Telfer B., Mandelli G.E., Santucci A., Vermi W., Giurisato E. (2020). Tumor-Associated Macrophages in Osteosarcoma: From Mechanisms to Therapy. Int. J. Mol. Sci..

[95] Siegel H.J., Pressey J.G. (2008). Current concepts on the surgical and medical management of osteosarcoma. Expert Rev. Anticancer.

[96] Marina N.M., Smeland S., Bielack S.S., Bernstein M., Jovic G., Krailo M.D., Hook J.M., Arndt C., van den Berg H., Brennan B. (2016). Comparison of MAPIE versus MAP in patients with a poor response to preoperative chemotherapy for newly diagnosed high-grade osteosarcoma (EURAMOS-1): An open-label, international, randomised controlled trial. Lancet Oncol..

[97] Wang Z., Wang Z., Li B., Wang S., Chen T., Ye Z. (2019). Innate Immune Cells: A Potential and Promising Cell Population for Treating Osteosarcoma. J. Cell. Biochem..

[98] Wedekind M.F., Wagner L.M., Cripe T.P. (2018). Immunotherapy for osteosarcoma: Where do we go from here?. Pediatr. Blood Cancer.

[99] Wan J., Zhang X., Liu T., Zhang X. (2016). Strategies and developments of immunotherapies in osteosarcoma. Oncol. Lett..

